# Atp6v1c1 May Regulate Filament Actin Arrangement in Breast Cancer Cells

**DOI:** 10.1371/journal.pone.0084833

**Published:** 2014-01-15

**Authors:** Shengmei Feng, Ming Cai, Pengcheng Liu, Li Wei, Jinshen Wang, Jin Qi, Lianfu Deng

**Affiliations:** 1 Shanghai Institute of Traumatology and Orthopaedics, Shanghai Key Laboratory for Prevention and Treatment of Bone and Joint Diseases with Integrated Chinese-Western Medicine, Ruijin Hospital, Jiao Tong University School of Medicine, Shanghai, China; 2 Department of Orthopaedics, Shanghai Tenth People's Hospital, Tongji University School of Medicine, Shanghai, China; Rutgers - New Jersey Medical School, United States of America

## Abstract

Previous studies have shown that the rate of breast cancer metastasis correlates with the expression of vacuolar H^+^-ATPases (V-ATPases). However, how V-ATPase is involved in breast cancer metastasis remains unknown. Our previous study showed that *Atp6v1c1*-depleted osteoclasts did not form organized actin rings and that Atp6v1c1 co-localizes with F-actin. In this study, we found that the normal arrangement of filamentous actin is disrupted in *Atp6v1c1*-depleted 4T1 mouse breast cancer cells and in the *ATP6V1C1*-depleted human breast cancer cell lines MDA-MB-231 and MDA-MB-435s. We further found that Atp6v1c1 co-localizes with F-actin in 4T1 cells. The results of our study suggest that high expression of Atp6v1c1 affects the actin structure of cancer cells such that it facilitates breast cancer metastasis. The findings also indicate that Atp6v1c1 could be a novel target for breast cancer metastasis therapy.

## Introduction

Studies of breast cancer have shown that vacuolar H^+^-ATPases (V-ATPases) located at the plasma membrane of highly metastatic human breast cancer cells are involved in the acquisition of a more metastatic phenotype [1]. In addition, the functional expression of plasmalemmal V-ATPases in drug-resistant human breast carcinoma cell lines is also involved in drug resistance [2]. On the basis of these findings, it is suggested that the V-ATPase complex is a potential target in breast cancer therapy and is an excellent candidate for anticancer drugs. However, how V-ATPase is involved in breast cancer metastasis remains unknown. Therefore, explaining the mechanism by which higher levels of V-ATPases correlate with increased breast cancer metastasis is very important, and it may reveal a specific target and candidate for a novel anticancer drug.

V-ATPases are composed of an ATP-hydrolytic domain (V1) and a proton-translocation domain (V0), as well as accessory subunits ac45 and M8–9 [3]. The V1 domain is located in the cytoplasm and contains 8 different subunits (A–H). The V0 domain, an integral membrane bound domain, consists of a, c, c′′, and d subunits in mammals [4], [5], [6]. The V-ATPase is a tightly coupled enzyme that exhibits activity only when the enzyme is fully assembled with all its subunits at the membrane; the C subunit is primarily responsible for its enzymatic function through the control of the reversible dissociation of the V0 and V1 domains [6], [7], [8], [9]. Subunit C was also reported to directly bind and stabilize filamentous actin (F-actin), increasing the initial rate of actin polymerization in a concentration-dependent manner and cross-linking actin filaments to bundles of varying thickness *in vitro* [10], [11]. Recently our study on osteoclasts (OC) also revealed that *Atp6v1c1*-depleted osteoclasts did not form organized actin rings and that Atp6v1c1 (C1) co-localizes with F-actin [12]. It is known that the dynamic regular arrangement of the actin cytoskeleton is important for tumor cell migration and invasion [13]. Moreover, in highly metastatic breast cancer cells (MB-231), V-ATPase colocalizes with actin at the cell surface, but it is inconspicuous in poorly metastatic breast cancer cells (MCF-7) [1]. Thereby, we hypothesized that subunit C interacts with the F-actin cytoskeleton and regulates F-actin rearrangement such that it facilitates breast cancer cell migration and invasion. To our knowledge, this is the first report that C1 deficiency can disrupt F-actin arrangement in breast cancer cells.

## Materials and Methods

### Cell lines and cell culture

MDA-MB-231 and MDA-MB-435s cells (American Type Culture Collection [ATCC], Manassas, VA) were cultured in DMEM supplemented with 10% fetal bovine serum (FBS), 1% l-glutamine, and 1% penicillin and streptomycin in 5% CO_2_ at 37°C. 4T1 cells (ATCC) were cultured in RPMI medium 1640 (Invitrogen, Carlsbad, CA) supplemented with 10% FBS, 1% l-glutamine, and 1% penicillin and streptomycin in 5% CO_2_ at 37°C.

### Preparation of pLB-c1s3 and pLB-LacZ lentiviruses and knockdown *Atp6v1c1* in 4T1 cells

Briefly, transfer vectors pLB-c1s3 or pLB-LacZ [12] were co-transfected with the packaging plasmids pCMV-Dr8.2 and pCMV-VSV-G (Addgene, Cambridge, MA) [14] into HEK293T cells by using a calcium phosphate co-precipitation method. The medium was replaced with fresh DMEM after co-transfection for 8 hours. The lentiviral supernatant was harvested after 48–72 hours, and titers were determined by infecting HEK293T cells with serial dilutions of concentrated lentivirus in the presence of 4 µg/ml polybrene (Sigma, St. Louis, MO). For depletion of C1, 4T1 cells were transduced with lentiviral supernatant for 8 hours, and the medium was replaced with fresh culture medium. Following this, cells were trypsinized 24 hours after infection and resuspended in culture medium. Single cell suspensions were seeded in 96-well culture plates. GFP^+^ monoclones expressing shRNA were observed as previously described [12]. We chose 4T1-LacZ as a control clone, and the 4T1-c1s3-1 and 4T1-c1s3-6 clones as *c1*-depleted 4T1 clones for further experiments.

### Preparation of RNA samples, reverse transcription-PCR (RT-PCR), and immunoblotting assays

These were performed as described in our previous study [12], [15]. Anti-Atp6v1c1 (H-300) was purchased from Santa Cruz Biotechnology (Santa Cruz, CA). Anti-tubulin (E7) was from the Developmental Studies Hybridoma Bank (DSHB, Iowa City, IA), and anti-β-actin monoclonal antibody (8H10D10) was purchased from Cell Signaling Technology (Danvers, MA). All assays were repeated 3 times.

### Cell staining for F-actin

Cells were fixed with 3.7% formaldehyde and permeabilized with 0.2% Triton X-100. The cells were then blocked with 1% goat serum and 3% bovine serum albumin (BSA) and incubated with 2 U/ml rhodamine phalloidin or 2 U/ml Oregon Green® 514 phalloidin (Molecular Probes, Carlsbad, CA) at room temperature for 20 minutes. Nuclei were visualized with 1 µg/ml 4′,6-diamidino-2-phenylindole (DAPI; Sigma). The experiment was performed in duplicate on 3 independent occasions in a 4-well chamber (Millipore, Billerica, MA).

### Cell immunofluorescence

Cells were grown in a 4-well chamber, fixed with 2% formaldehyde in phosphate-buffered saline (PBS) for 20 minutes, washed with PBS 3 times, incubated in 0.2% Triton X-100 for 15 minutes, and blocked for 1 hour with 10% normal donkey serum in PBS. Cells were incubated in the primary antibody (α-Atp6v1c1, 1∶100) diluted in 1% normal serum in PBS overnight at 4°C and then washed 3 times with PBS for 5 minutes and incubated with secondary antibody goat-anti-rabbit-FITC (1∶50) and rhodamine phalloidin (1∶100) for 1 hour. Cells were then washed with PBS, mounted with an anti-fade mounting medium containing DAPI, and then observed under a Zeiss LSM 510 confocal laser-scanning microscope (Zeiss, Germany) by using standard filter settings and sequential scanning to avoid crosstalk. The experiments were performed in triplicate.

### Knockdown of human ATP6V1C1 in MDA-MB-231 and MDA-MB-435s cell lines

Recombinant transfer vectors were from Sigma, expressing the puromycin resistance gene and driving shRNA expression from a human U6 promoter. A scrambled vector containing non-mammalian shRNA targets no known mammalian genes as a control; shRNA-1 and shRNA-2 containing shRNAs target human ATP6V1C1 mRNA (NM_001695), the targeting sequences are 5′-CCAAGACAGTTACCTGTGTA-3′ and 5′-CAATGCTACTTCAGCCCAA-3′, respectively. Each recombined transfer vector was co-transfected with the packaging plasmids pCMV-Dr8.2 and pCMV-VSV-G (Addgene) [14] into HEK293T cells using the calcium phosphate co-precipitation method. The medium was replaced with fresh DMEM after co-transfection for 8 hours. The lentiviral supernatant was harvested after 48–72 hours and MDA-MB-231 and MDA-MB-435s were infected with different lentiviral supernatants for 8 hours. The medium was replaced with fresh DMEM containing 10% FBS and 1 µg/ml puromycin for an additional 72 hours, and cells were harvested for western blot analysis or fixed for cell immunofluorescence. The experiments were performed in triplicate and for 3 times, independently.

## Results

### Knockdown of *Atp6v1c1* expression in 4T1 mouse breast cancer cells and lack of *Atp6v1c2* expression in Atp6v1c1-depleted 4T1 cells

**Figure 1:**
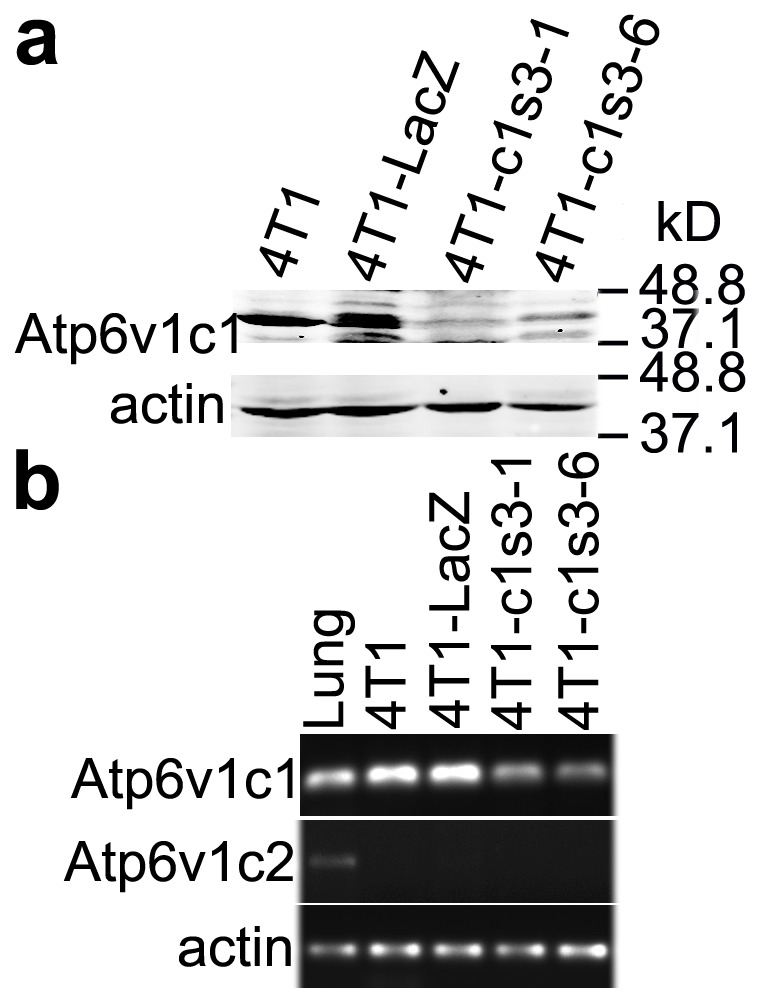
Atp6v1c1 knockdown 4T1 cell clone selection and lack of expression of *Atp6v1c2* in Atp6v1c1-depleted 4T1 cells. (a) Atp6v1c1 expression in different 4T1 cells as indicated by western blotting. (b) RT-PCR assays of lung and different 4T1 cells as indicated were performed with gene-specific primers for Atp6v1c1, Atp6v1c2, and β-actin.

To identify the role of the C subunit in F-actin arrangement in breast cancer cells, we knocked down C1 in 4T1 cells using lentivirus-mediated RNAi technology as previously described [12]. We found that C1 was significantly knocked down in the 4T1-c1s3-1 and 4T1-c1s3-6 cell clones compared to 4T1-LacZ (Fig. 1a,b). Meanwhile, C2 was not expressed in C1-knockdown 4T1 cells (Fig. 1b), which suggested that C2 did not compensate for the role of C1. We used mouse lungs as positive controls.

### Reducing Atp6v1c1 expression in 4T1 cells significantly impairs regular F-actin cytoskeleton arrangement

**Figure 2:**
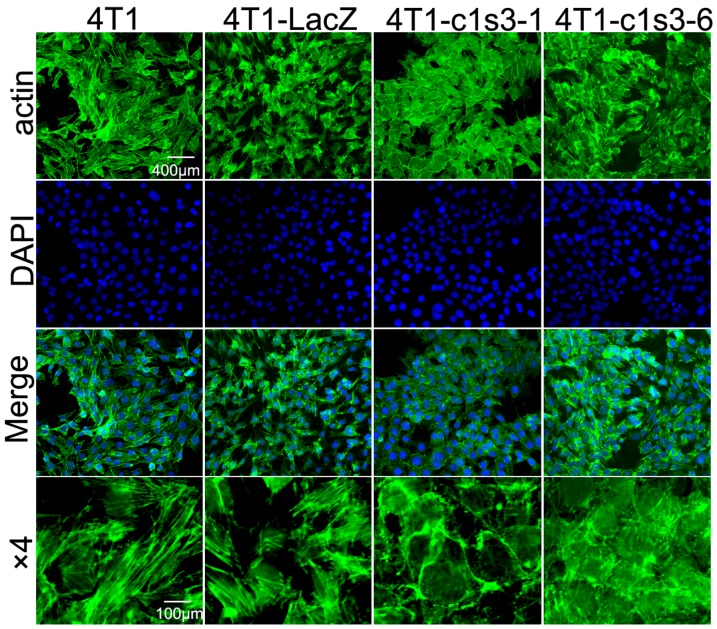
Regular F-actin arrangement was blocked in Atp6v1c1-depleted 4T1 cells. F-actin Oregon Green**®** 514 phalloidin staining of different 4T1 cells as indicated. Nuclei were visualized with DAPI. The cells shown are representative of the data (n = 3).

It was reported that tumor cell migration and invasion does not only depend on the proton pump function of plasma membrane V-ATPases, but also on other factors such as the dynamic regular arrangement of the actin cytoskeleton [13]. Recently, our study on osteoclasts revealed that C1 deficiency severely impaired mature osteoclast F-actin ring formation and that C1 colocalized with F-actin [12]. Therefore, we hypothesized that in addition to functioning as an essential subunit of V-ATPase, the C1 subunit regulates breast cancer cell F-actin arrangement, which may be responsible for increased plasma membrane V-ATPase expression correlating to higher breast cancer metastasis [1]. We performed F-actin staining (Fig. 2) and found that the actin cytoskeletons in both 4T1 and 4T1-LacZ cells displayed spear-shaped elongation in identical and regular orientation, which lead to cell stretching, suggesting that the cells were highly migratory and invasive [13]. The actin cytoskeletons in C1 knockdown 4T1-c1s3-1 and 4T1-c1s3-6 cells lost their identical and regular orientation, which led to cell rounding and suggested that the cells were minimally migratory and invasive. The results suggest that C1 is involved in the actin skeleton arrangement of breast cancer cells to facilitate tumor metastasis.

### Atp6v1c1 co-localized with F-actin in 4T1 cells

**Figure 3:**
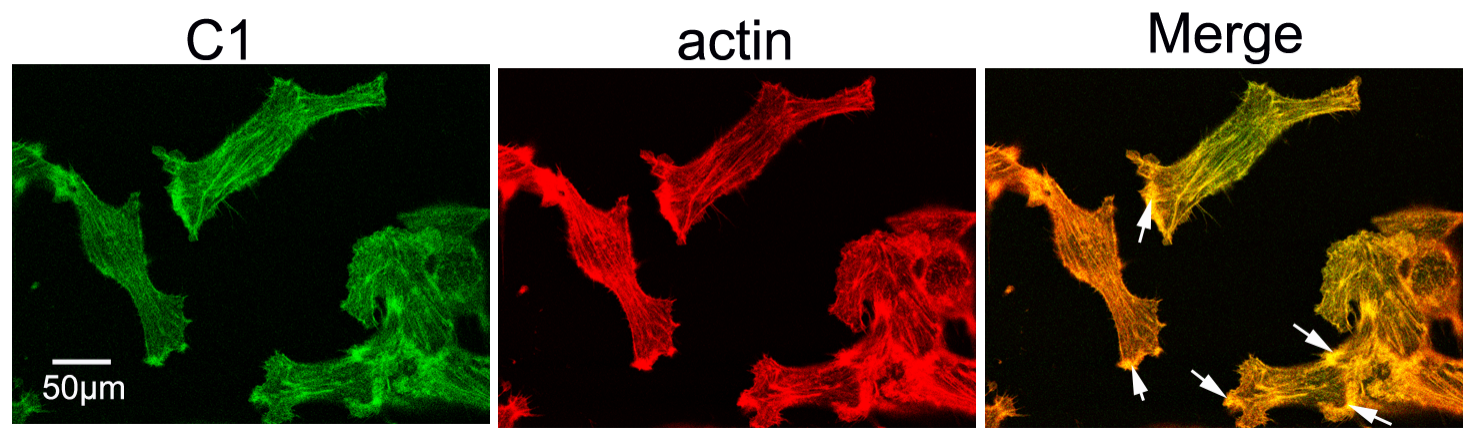
Atp6v1c1 co-localized with F-actin in 4T1 cells. Anti-Atp6v1c1 immunostaining and F-actin Oregon Green**®** 514 phalloidin staining of 4T1 cells. In the merged image, yellow staining showed that F-actin and Atp6v1c1 colocalized in the plasma and plasma membrane of 4T1 cells. The white arrows showed that most of the F-actin and Atp6v1c1 colocalization focused on the plasma membrane. The cells shown are representative of the data (n = 3).

To clarify whether C1 also interacts with F-actin in breast cancer cells, we performed cell immunofluorescence and viewed the images using a confocal laser-scanning microscope. We found that C1 and F-actin co-localized in 4T1 cells (yellow staining in Merge; Fig. 3), and a large amount of their colocalization focused on the plasma membrane of the cell (white arrows; Fig. 3). Together, this data further supports the hypothesis that C1 regulates actin cytoskeleton arrangement in breast cancer cells.

### Knockdown of *ATP6V1C1* expression in the human breast cancer cell lines MDA-MB-231 and MDA-MB-435s blocks the regular arrangement of the cell actin cytoskeleton

**Figure 4:**
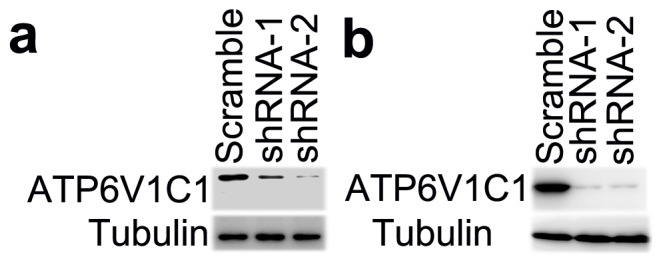
Verification of shRNA targeting to human ATP6V1C1 in human breast cancer cell lines. (a) ATP6V1C1 expression in different lentivirus-treated MDA-MB-231 cells as indicated by western blotting. (b) ATP6V1C1 expression in different lentivirus-treated MDA-MB-435s cells as indicated by western blotting.

**Figure 5:**
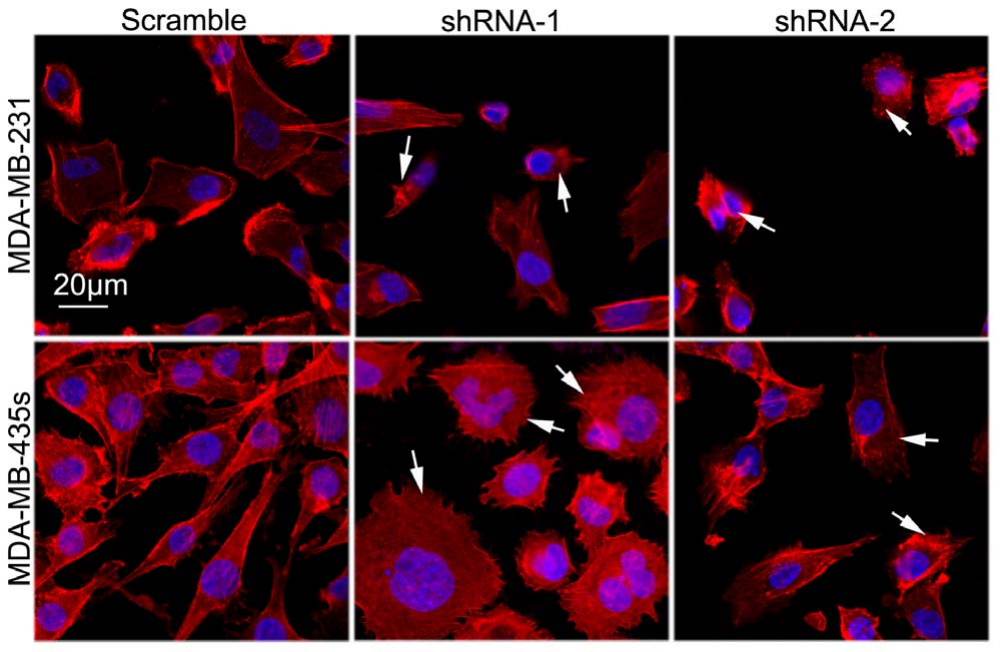
Regular F-actin arrangement was blocked in ATP6V1C1-depleted MDA-MB-231 and MDA-MB-435s cells. F-actin rhodamine phalloidin of different lentivirus-treated cells as indicated. The white arrows showed cells with disrupted actin cytoskeleton compared to control cells. The cells shown are representative of the data (n = 3).

To further confirm the role of the C subunit in F-actin arrangement in breast cancer cells, we knocked down C1 in human breast cancer cells MDA-MB-231 and MDA-MB-435s as described previously [12]. We found that both shRNA-1 and shRNA-2 can significantly knocked down C1 expression in MDA-MB-231 (Fig. 4a) and MDA-MB-435s (Fig. 4b) cells compared to scrambled shRNA. We then analyzed the actin of different shRNA lentivirus-treated cells, and found that both in shRNA-1 and shRNA-2 treated MDA-MB-231 and MDA-MB-435s (Fig. 5) cells, the regular arrangement of actin as seen in the scrambled shRNA control treated cells was lost (most of them lined the plasma membrane) (Fig. 5). Together, our results suggest that C1 also regulates actin cytoskeleton arrangement in breast cancer cells besides acting as a regulator of V-ATPase to facilitate breast cancer metastasis. Our results suggest a new mechanism by which higher levels of plasma membrane V-ATPase correlates to a more metastatic phenotype in human breast cancer cells [1].

## Discussion

V-ATPases located at the plasma membrane of highly metastatic human breast cancer cells are involved in the acquisition of a more metastatic phenotype [3]; nonetheless, the exact mechanism remains unknown. ATP6V1C1, an essential subunit of V-ATPase and a regulator of V-ATPase activity [6], [7], [8], [9], was recently reported to be the most strongly overexpressed gene in oral squamous cell carcinoma compared to other subunits of V-ATPase, and it was suggested to control tumor growth and metastasis [16]. Recently, it was reported that subunit C can increase the initial rate of actin polymerization and directly bind, stabilize, and cross-link F-actin [10], [11], and growing number of evidence supports that dynamic F-actin arrangement plays an important role in tumor invasion [13], [17]. Together, these results lead us to wonder whether the C1 subunit is involved in actin arrangement in breast cancer cells to facilitate tumor metastasis.

To determine the role of subunit C1 breast cancer cells actin cytoskeleton arrangement, we used lentivirus-mediated siRNA to knockdown C1 expression in 4T1 cells and selected 2 *c1*-knockdown 4T1 cell clones. We found that C2, another isoform of the C subunit, did not express in *c1*-knockdown 4T1 cells, which excludes a compensatory effect of C2 for the role of the C subunit. We checked actin arrangement in different lentivirus-treated 4T1 cells and found that reducing C1 expression in 4T1 cells significantly impairs regular actin cytoskeleton arrangement. Furthermore, we found that C1 co-localized with actin in 4T1 cells and a large amount of their colocalization focused on the plasma membrane of the cell. This data is consistent with the report that the colocalization of V-ATPases with actin at the surface of breast cancer cells was linked to higher metastatic activity [1]. Notably, we further confirmed the role of C1 in actin arrangement in human breast cancer cell lines MDA-MB-231 and MDA-MB-435s. Taken together, these data suggest that in addition to being an essential component of V-ATPases, C1 is involved in dynamic actin cytoskeleton arrangement that facilitates tumor cell metastasis when it is expressed at high levels. Recently, it was discovered that the a3 and a4 isoforms play a significant role in invasion by affecting the pH of the cytosol and intracellular compartments and by targeting V-ATPases to the plasma membrane of MB231 cells, respectively [18], which, combined with our results, helps elucidate how V-ATPases located at the plasma membrane of highly metastatic human breast cancer cells are involved in the acquisition of a more metastatic phenotype [1]. Indeed, we have done the wound healing assay and matrigel invasion assay in 4T1 cells, and found that knock-down of Atp6v1c1 in 4T1 cells can significantly reduce cells migration and invasion. We have reported our results in the ASMBR 2010 Annual Meeting as a poster, poster number was SA0127 and the title was “Inhibition of Atp6v1c1 (a Subunit of the V-ATPase) Expression Decreases 4T1 Mouse Breast Cancer Growth, Invasion and Osteolytic Lesion”.

The results of our present study show, for the first time, that in addition to an essential subunit of the V-ATPase and thereby playing a role in breast cancer growth and metastasis, the C1 subunit play an important role in breast cancer metastasis, i.e., the manipulation of F-actin cytoskeleton arrangement so that it facilitates cancer cell metastasis. These results provide new evidence for the important role of plasma membrane ATPases, such as the Na^+^/K^+^-ATPase [19] and the V-ATPase [20], in breast cancer metastasis. Our results suggest that C1 would be a useful target for inhibiting metastasis of breast cancer and even other cancers such as oral squamous cell carcinoma [16] with high C1 levels.
