# Correction: Atp6v1c1 May Regulate Filament Actin Arrangement in Breast Cancer Cells

**DOI:** 10.1371/annotation/ca9f2d5b-93ab-4d7c-b577-cd045b343e53

**Published:** 2014-01-30

**Authors:** Ming Cai, Pengcheng Liu, Li Wei, Jinshen Wang, Jin Qi, Shengmei Feng, Lianfu Deng

The order of authors is incorrect. Please view the correct author order, order of affiliations and Citation below:

Ming Cai^1^, Pengcheng Liu ^1^, Li Wei ^2^, Jinshen Wang ^2^, Jin Qi ^2^, Shengmei Feng ^2^, Lianfu Deng ^2^

1 Department of Orthopaedics, Shanghai Tenth People’s Hospital, Tongji University School of Medicine, Shanghai, China

2 Shanghai Institute of Traumatology and Orthopaedics, Shanghai Key Laboratory for Prevention and Treatment of Bone and Joint Diseases with Integrated Chinese-Western Medicine, Ruijin Hospital, Jiao Tong University School of Medicine, Shanghai, China

Cai M, Liu P, Wei L, Wang J, Qi J, et al. (2014) Atp6v1c1 May Regulate Filament Actin Arrangement in Breast Cancer Cells. PLoS ONE 9(1): e84833. doi:10.1371/journal.pone.0084833

